# Preclinical evaluation of safety and potential of black hellebore extracts for cancer treatment

**DOI:** 10.1186/s12906-019-2517-5

**Published:** 2019-05-21

**Authors:** Jennifer E. Felenda, Claudia Turek, Nora Mörbt, Anja Herrick, Margit B. Müller, Florian C. Stintzing

**Affiliations:** 1 Pharmacological and Clinical Research, WALA Heilmittel GmbH, Dorfstr. 1, 73087 Bad Boll, Eckwälden Germany; 2 Drug Safety/Information, WALA Heilmittel GmbH, Dorfstr. 1, 73087 Bad Boll, Eckwälden Germany; 3 Analytical Development & Research, WALA Heilmittel GmbH, Dorfstr. 1, 73087 Bad Boll, Eckwälden Germany

**Keywords:** *Helleborus niger*, Ranunculaceae, Saponin, HUVEC, Tumor cell, Non-mutagenic, Anti-cancer, Anti-angiogenetic, Anti-proliferative, Hemolytic activity

## Abstract

**Background:**

The therapeutic use of *Helleborus niger* L. is manifold due to its specific phytochemical composition. Two compound groups, the ranunculin derivates including protoanemonin and the steroidal saponins, are also associated with toxicity (genotoxicity, disintegration of membrane structures). Therefore, in vitro investigations were performed on safety aspects of a *Helleborus niger* aqueous fermented extract (*HNE*). In addition its therapeutic potential against various cancer cell lines was assessed to gain insight into the respective mechanisms of action.

**Methods:**

To evaluate the safe use of *HNE*, Ames and hemolytic tests were carried out. Two angiogenesis assays in 2D and 3D design were conducted to assess the anti-angiogenetic potential, for which human umbilical vein endothelial cells (HUVEC) were chosen. A panel of tumor cell lines was used in 2D and 3D proliferation assays as well in the migration- and invasion-assay. All investigations were performed with *HNE* compared to reference substances. The 2D proliferation assay was additionally performed with isolated compounds of *HNE* (characteristic steroidal saponins).

**Results:**

*HNE* did not exhibit any genotoxic potential. Concentrations up to 10 μl/ml were classified as non-hemolytic. *HNE* exerted anti-angiogenetic effects in HUVEC and anti-proliferative effects in five cancer cell lines, whereas hellebosaponin A and D as well macranthosid I did not show comparable effects neither singly nor in combination. Due to the inherent instability of protoanemonin in isolated form, parallel investigations with protoanemonin could not be performed. *HNE* (600–1000 μg/ml) inhibited the migration of certain cancer cells by > 80% such as Caki-2, DLD-1 and SK-N-SH.

**Conclusion:**

*HNE* exhibit neither genotoxic nor hemolytic potential. The present investigations verify the anti-angiogenetic effects on HUVEC, the anti-proliferative effects and migration-inhibiting properties on tumor cells. The lower effect of the relevant steroidal saponins compared to the whole extract underlines the fact that the latter is more effective than a blend of isolated pharmacologically active components.

**Electronic supplementary material:**

The online version of this article (10.1186/s12906-019-2517-5) contains supplementary material, which is available to authorized users.

## Background

**Fig. 1:**
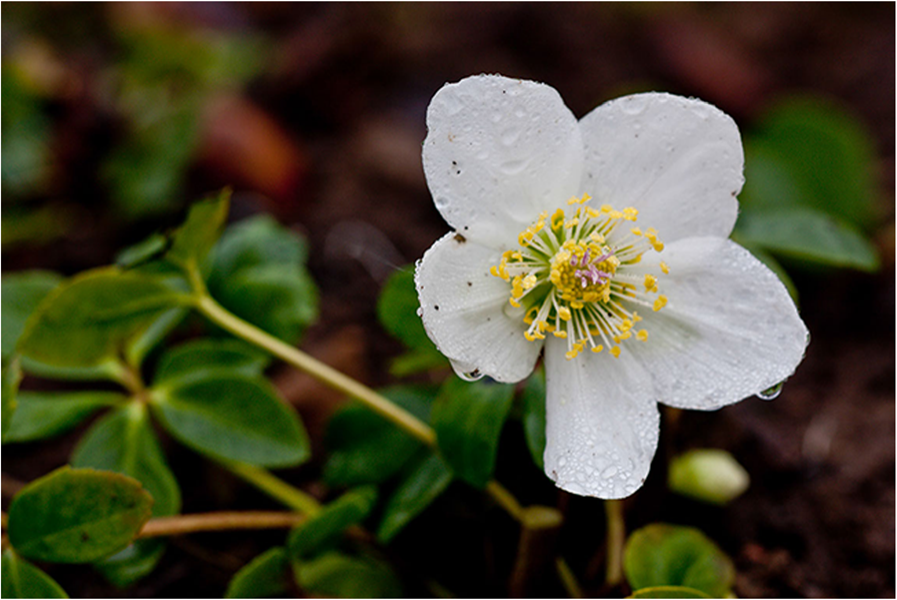
*Helleborus niger* L. Black hellebore is a perennial herb with a shallow rhizome blossoming during winter. Taxonomically it is grouped with the Ranunculaceae. Black hellebore has been used as a remedy since ancient times. Because of the multiple components, a broad pharmacological spectrum is ascribed to Christmas rose: anti-bacterial, anti-inflammatory, cholesterol- and blood glucose-lowering, neuroprotective, hepatoprotective and immune-modulating effects. With respect to pharmacologically active ingredients, four compound classes are particularly relevant, i.e. steroidal saponins, ranunculin derivatives (including protoanemonin), beta-ecdysone and flavonoids. Photograph was taken by WALA Heilmittel GmbH

Black hellebore (*Helleborus niger* L. (*H. niger*)), also referred to as Christmas rose, is a perennial herb with a shallow rhizome blossoming during winter. Taxonomically it is grouped with the Ranunculaceae and can be found in the woods in mountain regions including Germany at 400–1800 m above sea level (Fig. 1) [1, 2].

Black hellebore has been used as a remedy since ancient times. Paracelsus (1493–1541) knew about the healing power of the Christmas rose and recommended the leaves for prevention of dementia and the root extract was applied against gouty arthritis and other joint diseases, epilepsy, apoplexy, kidney weakness with edema and gynecological complaints. Samuel Hahnemann (1755–1843) reported *H. niger* as a remedy to treat depression, lethargy and sleepiness. Rudolf Steiner (1829–1910) assumed an anti-tumor activity [3]. Today, a broad pharmacological spectrum is ascribed to Christmas rose because of its multitude of compounds: anti-bacterial, anti-inflammatory, cholesterol- and blood glucose-lowering, neuroprotective, hepatoprotective and immune-modulating effects [2, 4].

Preparations from the complementary medicine portfolio containing black hellebore are used in the concomitant therapy of oncological diseases. Therefore, the aim of the second part of this investigation was to obtain a better understanding of the therapeutic potential of *Helleborus niger* extract *(HNE)* in vitro. The main clinical indications are brain tumors and lymphomas [5]. Furthermore *H. niger* is related to the renal system because of its diuretic effect and its ability to support elimination of edemas through kidney activation [6].

Black hellebore has only recently regained scientific interest as a plant exhibiting an interesting multi-component profile [1, 2, 4–6].With respect to pharmacologically relevant ingredients, four compound classes are in the forefront, i.e. the steroidal saponins, the ranunculin derivatives (including protoanemonin), beta-ecdysone and the flavonoids [4, 7, 8]. The latter two exert anabolic and cell protective actions [9, 10] while saponins possess the ability to disintegrate membrane structures [11–13]. Protoanemonin is an unsaturated lactone that is formed by deglycosylation from ranunculin, its precursor. Protoanemonin as such is a caustic compound released following comminution of *Helleborus* plant material and – despite its strong tendency to dimerize when occurring as an isolated compound – appears to be fairly stable in aqueous media (unpublished data). Black hellebore, in particular its constituent protoanemonin, has sporadically been suspected to induce genotoxicity, but also anti-mutagenic and even anti-leukemic properties have been reported [14–16]. Though rarely occurring, a large number of the symptoms from poisoning with black hellebore described in the scientific literature can be attributed to the membranolytic properties of some steroidal saponins present in both underground and aerial parts of the plant [17, 18]. Inter alia, this is expressed by hemolytic activity, hence a potential to disintegrate the cellular membranes of red blood cells [19]. A thorough update of the steroidal saponins with their amphiphilic structures has recently been provided [4, 7, 8]. Among the 38 structures assigned, hellebosaponins A and D were prevailing. This is in contrast to previous reports where macranthosid I was proposed to be the dominating compound [4]. While the roots are especially rich in acetylated polyhydroxy-saponins [8], the aerial parts were characterized by the presence of sarsasapogenyl- and diosgenyl structures [4]. Due to their high solubility in hydrophilic media and their decent stability in isolated conditions, the focus of the present investigation was on the saponins. As in *HNE*, a conversion of the genuine saponin profile proceeds [7], an evaluation of the pharmacological potential of both isolated reference substances and the aqueous fermented extract appeared to be of interest. A causal relationship between compound quantity and pharmacological effect of multi-compound blends cannot be easily drawn. Therefore, by comparison of the complete extract with isolated compounds, the potential of inhibiting cancer cell metabolism and consecutive anti-proliferative effects on living cell systems were investigated. Furthermore the anti-angiogenetic characteristics on HUVEC as well as the anti-proliferative and non-migration properties of *HNE* on cancer cells were elaborated. The results obtained from these preclinical investigations shall help to better understand the actions of black hellebore on healthy and pathological cell systems and shall deepen our understanding into the therapeutic potential of *H. niger* preparations, especially for the treatment of oncologic events.

## Methods

Table 1 shows an overview of the assays used as a basis for the therapeutic potential of *HNE*. The most important conditions of the assays (cell types, incubation time, reference substances, minimal and maximal concentration of test item) are compiled. The two investigations to evaluate the safety potential of *HNE* are not included.

**Table 1:** Overview of the assays used to evaluate the therapeutic potential of *HNE*

Assay	Test principle	Cells	Incubation time (h)	Reference substance	Max. Conc. (μg/ml)	Min. Conc. (μg/ml)
WST-1	Cell viability	HUVEC	24	Paclitaxel	50,000	625
BrdU	Cell proliferation	HUVEC	24	Paclitaxel	50,000	625
Matrigel Angiogenese	Tube Formation 2D	HUVEC	24	Untreated cells	829	13
Spheroid-based Cellular 3D Angiogenesis	Tube Formation 3D	HUVEC	24	Sunitinib	20,000	20
Alamar Blue	Cell viability 2D	Tumor cells	72	Actinomycin-D	10,000	3.33
Soft Agar	Cell viability 3D	Tumor cells	216–288	Actinomycin-D	10,000	3.33
Oris™	Cell migration and invasion	Tumor cells	24–48	SKI-606 (Bosutinib)	1000	600

Seven in vitro assays were used to investigate the mechanism of action and the therapeutic potential of *HNE* for tumor therapy

### *Helleborus niger* extract (HNE)

*Helleborus niger* L. plant material was obtained from our company-owned (WALA Heilmittel GmbH) medicinal herb garden, which is located in Bad Boll/Eckwaelden in Southwestern Germany. The cultivation site and identification procedure for plant material used have been described previously [4]: The cultivation areas are maintained following the principles of biodynamic agriculture and organic farming. *H. niger* L. plants were harvested by botanically well-trained personnel in winter (January). Botanical identification of plant material was carried out by trained staff according to the principles of GACP (good agricultural collection practice), i.e. according to valid requirements for the manufacturing of medicinal products in Europe. Preparation of aqueous-fermented extract of the fresh whole flowering plant was carried out according to the official production protocol described in the German Homeopathic Pharmacopoeia (GHP), aqueous-fermented mother tinctures, protocol number 34c [20]. Two representative batches of *HNE* (A and B) were used. Batch A contained 4020.5 μg/ml total saponins, 34% of overall saponin content was hellebosaponin A and 18% hellebosaponin D, respectively. The content of total saponins in batch B was lower (2943.0 μg/ml), but the percentage of hellebosaponin A was even higher (45%). Contents of protoanemonin were 174 μg/ml (batch A) and 90 μg/ml (batch B). All dilutions of *HNE* were performed with isotonic solution (contains 0.88% NaCl and 0.02% NaHCO_3_; WALA Heilmittel GmbH) except for the reverse mutation assay, where purified water was used (BSL Bioservice, Planegg, Germany).

### HPLC-DAD analysis of saponins and protoanemonin

**Fig. 2:**
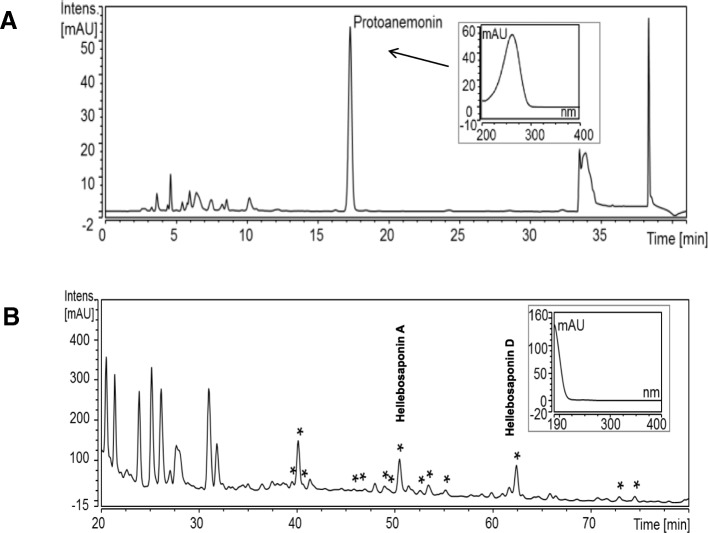
HPLC-DAD of an aqueous fermented *Helleborus niger* extract. **a**: HPLC-DAD chromatogram (λ = 261 nm) of an aqueous fermented *Helleborus niger* extract (1:20 dilution in water) and UV/Vis spectrum of protoanemonin. **b**: HPLC-DAD chromatogram (λ = 210 nm) of an aqueous fermented *Helleborus niger* extract and exemplary UV/Vis spectrum of a saponin. * Saponins

Quantification of saponin and protoanemonin contents was carried out by high pressure liquid chromatography with diode array detection (HPLC-DAD) using a SunFire™ C18 (150 × 2.1 mm i.d., 5 μm particle size; Waters GmbH, Eschborn, Germany) column for saponin and a Reprosil-AQ reversed phase column (250 × 4 mm i.d., 5 μm particle size, Maisch, Ammerbruch, Germany) for protoanemonin analysis, respectively (Fig. 2). α-angelica lactone (PhytoLab GmbH & Co. KG, Vestenbergsgreuth, Germany; purity 79%) was used as analytical reference for quantification of protoanemonin and the total saponin content was quantified at 210 nm using hederacoside C (PhytoLab GmbH & Co. KG, Vestenbergsgreuth, Germany; purity 88%) as a reference. Gradient elution was carried out for both analyte groups, and detection wavelengths were 210 nm (saponins) and 261 nm (protoanemonin). Further details on both HPLC methods as well as method validation data have been reported earlier [21].

### Isolated steroidal saponins

Hellebosaponin A, hellebosaponin D and macranthosid I were isolated and purified according to [8]. Chromatographic purity of hellebosaponin A and hellebosaponin D was 97 and 88% for macranthosid I by high pressure liquid chromatography (HPLC). Isotonic solutions were prepared therefore containing 1.31, 0.64, and 0.12 mg/ml, respectively. Test solutions with isolated steroidal saponins were derived from average contents of 9 different *HNE* batches. The concentrations employed were representative for their respective content in the *HNE* batches applied.

### Reference substances

Paclitaxel (LC Laboratories, Boston, USA) was used as the reference substance in the Water soluble tetrazolium-assay (WST-1) and Bromodeoxyuridine-assay (BrdU). Its purity was > 99% by HPLC.

Sunitinib (LC Laboratories, Woburn, USA) was used in the Spheroid-based cellular 3D Angiogenesis assay. Its purity was > 99% by HPLC.

Actinomycin-D (Sigma-Aldrich Chemie GmbH, Munich, Germany) served as reference substance in the Alamar Blue and Soft Agar assays. Its purity was ≤ 98% by HPLC.

SKI-606 or Bosutinib (Selleckchem, Munich, Germany) was used in the Oris™ Cell Migration and Invasion assay and exhibited a purity of > 99.9% by HPLC.

### HUVEC and cancer cell lines

In the Alamar Blue and in the Soft Agar assays five cancer cell lines were investigated: Caki-2 (renal cell cancer), MKN-1 (gastric cancer), DLD-1 (colorectal cancer), SK-N-SH (neuroblastoma) and LN 229 (glioblastoma). Additionally, HeLa (cervical carcinoma), HCC827 (lung cancer), A375 and A431 (melanoma), DU-145 (prostate carcinoma), SK-OV3 (ovarian cancer), MDA-MB-231 and MCF-7 (breast cancer) as well as U87-MG (glioblastoma) were tested in the Oris™ Cell Migration and Invasion assay. All tumor cell lines were provided by ProQinase GmbH (Freiburg, Germany). MKN-1-, HCC827- and A431-cells were cultivated in RPMI-1640 (Rosewell Park Memorial Institute) containing 10% FCS (Fetal Calf Serum) and Penicillin / Streptomycin. All remaining cells were kept in DMEM (Dulbecco’s Modified Eagle’s Medium) containing 10% FCS and Penicillin / Streptomycin at 37 °C and 10% CO_2_, respectively. A pooled source of Human umbilical vein endothelial cells (HUVEC) was purchased from Promocell (Heidelberg, Germany). These cells are generally used for in vitro angiogenesis assays. HUVEC were cultivated in ECGM (Endothelial Cell Growth Medium) and ECBM (Endothelial Cell Basal Medium) with 20% FCS for 24 h at 37 °C, 5% CO_2_ and 95% humidity prior to addition of individual test items.

### Safety assessment

Two preclinical investigations to improve the safety profile of *H. niger* were carried out in compliance with good laboratory practice.

#### Reverse mutation assay (Ames test)

Tests with *HNE* were performed by BSL Bioservice Scientific Laboratories GmbH (Planegg, Germany) according to Organisation for Economic Cooperation and Development (OECD) guidelines [22]. Specified tester strains of *Salmonella typhimurium* TA98, TA100, TA1535, TA1537, and TA102 were used in the plate-incorporation and the pre-incubation method, respectively. Both tests were performed with and without metabolic activation using mammalian microsomal fraction Supernatant 9000 x g (S9) mix preparation according to Ames et al. [23] or S9 mix substitution phosphate buffer (0.2 M), respectively. For each experiment, 100 μl of several dosing levels of *HNE* diluted in purified water up to the maximum concentration according to recommendations from the OECD guideline [22] and concentration finding experiments in a pre-experiment for toxicity evaluation were applied (0.0316, 0.100, 0.316, 1.0, 2.5 and 5.0 μl/ plate). Purified water was used as negative control and strain-specific positive controls were applied in parallel [20]. Samples including negative and positive controls were tested in triplicate. Results were evaluated for cytotoxic and mutagenic effects using criteria described by OECD [22].

#### Hemolytic potential

The hemolytic activity on human erythrocytes was investigated by Harlan CCR (Rossdorf, Germany) according to the design of the International Organization for Standardization (ISO) 10993 guideline. Undiluted *HNE*, dilutions thereof at concentrations ranging from 100 to 0.001 μl/ml (in intervals of 1:10), each in isotonic solution, were treated with erythrocyte suspension while being shaken for 3 h at room temperature in the dark. Concentrations were chosen taking into account the concentrations used in medical treatment. Erythrocytes were obtained from donor blood free from medication. The diluted blood was centrifuged for 5 min at 2000 rpm, the supernatant discarded and the erythrocyte pellet washed three times with physiological sodium chlorine solution. Subsequently, the washed cells were re-suspended in saline such that 25 μl of the suspension in 1 ml of de-ionized water resulted in an absorbance of 2.1 at 575 nm. Saline and de-ionized water, serving as negative and positive controls respectively, were equally treated. After centrifugation at about 3000 x g for 1 min, absorbance of the supernatants derived from treated erythrocytes was measured at a wavelength of 530 nm. Absorbance values analyzed subsequent to erythrocyte treatment were corrected with absorbance values from untreated test items. Hemolysis was reported as the percentage of the hemoglobin liberated from the erythrocytes, measured as mean absorbance values corrected by absorbance values of untreated test items, as compared to positive control (100% lysis).

### Anti-angiogenesis and tube formation

Angiogenesis, the formation of new blood vessels from pre-existing ones, is a balanced physiological process during growth and development. Among others, angiogenesis is also mandatory for invasive tumor progression. Therefore, the anti-angiogenetic impact of *HNE* was tested.

#### WST-1

Prior to performing the Matrigel Angiogenesis assays, the cytotoxic activity of *HNE* on HUVEC was investigated by a colorimetric assay. Cell viability was quantified by transformation of the tetrazolium salt WST-1. Cells in supplemented cell culture medium were plated (2.5 × 10^3^ per well) in a volume of 100 μl and incubated for 24 h at 37 °C, 5% CO_2_ and 95% humidity prior to the addition of test items. Cells cultured with medium alone served as negative control. Paclitaxel in DMSO was tested as reference item in final concentrations of 8539.06–0.0085 μg/ml. The following dilutions of *HNE* were used: 1:20, 1:100, 1:500, 1:2500, 1:12,500, 1:62,500 and 1:312,500 according to previous investigations [2, 5, 6]. Each test item in each dilution was analyzed in triplicate. Cells were incubated in the presence of test items for 24 h. WST-1 (cell proliferation reagent WST-1, supplied as ready-to-use solution, Roche Molecular Biochemicals, Mannheim, Germany) was added (20 μl per well), incubated at 37 °C and the optical density (OD) was determined after 4 h. OD was measured in an ELISA reader (FluoStar Optima, BMG Lab-tech, Germany) at wavelength settings of 450 nm and 645 nm. OD_645nm_ was subtracted from the OD_450nm_ to correct for background.

#### BrdU

The second step was to evaluate the anti-proliferative efficacy of *HNE* in dilutions similar to WST-1 and to define the concentration of *HNE* to be used in the Matrigel Angiogenesis assay.

A chemiluminescent cell proliferation assay (Roche Diagnostics Deutschland GmbH, Mannheim, Germany) was performed according to the manufacturer’s instructions. *HNE* was diluted in 1:20, 1:100, 1:500, 1:2500, 1:12,500, 1:62,500 and 1:312,500 according to previous investigations [2, 5, 6]. Paclitaxel was tested as positive reference in concentrations of 8539.06–0.0085 μg/ml. In addition, cells cultured with medium alone served as negative control. Cells were incubated for 24 h. Luminescence was measured in an enzyme-linked immunosorbent assay (ELISA) reader FluoStar Optima (BMG Labtech, Ortenberg, Germany). Each test item in each dilution was tested in triplicate.

#### Matrigel angiogenesis assay

This test monitors the ability of HUVEC to form capillary-like structures (tubes) in 2D in the presence of added dilutions at non-cytotoxic levels assessed in the WST-1. This in vitro method was performed as described by Ponce [24] with minor modifications by HeidelbergPharma GmbH (Heidelberg, Germany). HUVEC with ECGM, supplement mix (Promocell, Heidelberg, Germany) and 20% heat inactivated FCS in the matrigel-well were incubated in duplicate with *HNE* dilutions that had been previously determined in BrdU as inhibitory concentration of 10% (IC_10_) (batch A: 13 μg/ml; batch B: 35 μg/ml), inhibitory concentration of 50% (IC_50_) (batch A: 69 μg/ml; batch B: 170 μg/ml) and inhibitory concentration of 90% (IC_90_) (batch A: 354 μg/ml; batch B: 829 μg/ml). After 24 h, cells were labeled with 8 μg/ml Calcein AM Fluorescent Dye (Bectin Dickinson, Heidelberg, Germany) and examined under a microscope. A digital photograph was taken and the cell-covered area (in μm^2^) in each representative area (ROI) was determined.

#### Spheroid-based cellular 3D angiogenesis assay

Verifying the results of the in vitro Matrigel Angiogenesis assay, a second angiogenesis assay in 3D dimension was performed by ProQinase GmbH (Freiburg, Germany). Experiments were conducted in modification of the originally published protocol by Korff and Augustin [25]. *HNE* was tested for its ability to inhibit vascular endothelial growth factor A (VEGF-A) induced endothelial cell (EC) sprouting. Spheroids are prepared by pipetting 500 HUVEC in a hanging drop on plastic dishes to allow overnight spheroid aggregation [26]. Fifty spheroids were seeded in a collagen solution to allow collagen gel polymerization. Test items (*HNE* diluted in 1:50, 1:167, 1:500, 1:1667, 1:5000, 1:16,667 and 1:50,000 based on results from concentrations used in the 2D Angiogenese assay), Sunitinib (3984.8–3.99 μg/ml) and hVEGF-A_165_ (ProQinase GmbH, Freiburg, Germany) with a final assay concentration of 25 ng/ml were added on top of the polymerized gel. Following incubation for 24 h, plates were fixed by adding 4% Roti-Histofix (Roth, Karlsruhe, Germany). Sprouting intensity of EC spheroids is quantified by determining the cumulative sprout length per spheroid using an inverted microscope and digital imaging software analysis (Soft imaging system, Muenster, Germany). The mean of the cumulative sprout length of 10 randomly selected spheroids is considered as an individual data point. The median of basal sprouting was subtracted from all other median data points.

### In vitro assessment of anti-cancer potential

The following three in vitro assays, performed by ProQinase GmbH (Freiburg, Germany), were applied to assess the impact of *HNE* on different human cancer cell lines.

#### Proliferation-assays: Alamar blue and soft agar assays

Both the 2D Alamar Blue and the 3D Soft Agar assay are based on the quantification of the population of living cells after compound incubation using a fluorescent cell viability dye. The test items were *HNE* at a dilution of 1:100 with further semi-logarithmic dilution steps according to the standard concentration range in these assays: isotonic solution, Actinomycin-D (< 98% purity) and Staurosporine (Sigma-Aldrich Chemie GmbH, Munich, Germany) (purity > 95% by HPLC). In the 2D Alamar Blue assay the following isolated compounds from *H. niger* were investigated: 0.12 mg/ml macranthosid I, 1.31 mg/ml hellebosaponin A, 0.64 mg/ml hellebosaponin D as well as the combination of these three steroidal saponins. The incubation time was 72 h in the 2D Alamar Blue assay and 9 days for DLD-1, SK-N-SH, MKN-1 and LN229 as well as 12 days for Caki-2 in the 3D Soft Agar assay. Each sample was submitted to three independent experiments.

#### Migration assay

Cancer cells are able to migrate, intrude into other tissues and form metastases. *HNE* was tested for its impact on migration of human tumor cell lines. For this purpose, the Oris™ Cell Migration and Invasion assay was used. Cells were seeded (40,000 cells in 100 μl of medium/well) onto Collagen-I-coated 96-well plates (ORIS™, AMS Bio, Cambridge, UK). Vials were equipped with stoppers to restrict cell seeding to the outer annular regions of the well. Removal of the stopper 18 h after seeding reveals an unseeded region in the center of each well, i.e. the detection zone, into which the seeded cells then may migrate during 24–48 h in the absence or presence of inhibitors. *HNE* was added after removing the stopper at concentration levels chosen by means of the results from all previous tests: 600 μg/ml for batch A and 1000 μg/ml for batch B. This is roughly equivalent to the homeopathic potency D3. All concentration levels of *HNE* were monitored in triplicate. Zero control (with stopper inserts) was carried out 4-fold and positive control (solvent) 5-fold. SKI-606 was used as reference compound and assessed at six different concentration levels in singlicate (5304.46–0.053μg/ml). Fluorescence data was determined using fluorescence plate reader. The mean value of solvent controls was set to 100% and the mean value of zero control was set to 0%.

### Statistical analysis

All standard deviations (SD) are shown in the Additional file 1.

GraFit Version 5.0.13 from Erithacus Software Ltd. (West Sussex, U. K.) was used for calculating of IC_50_ in WST-1 and BrdU.

In the Matrigel Angiogenesis assay the cell-covered area in untreated control wells (mean of 6 ROI in 2 wells) and test item-treated wells (mean of 6 ROI in 2 wells) were compared.

Raw data were converted into percent EC sprouting relative to high control (VEGF-A sprouting) which was set to 100% for the spheroid-based Cellular 3D Angiogenesis assay and into percent cell viability relative to high (solvent) and low (Staurosporine), which were set to 100 and 0% for the proliferation assays, respectively. For these methods GraphPadPrism Version 5 from GraphPad Software (La Jolla, USA) was used for calculating IC_50_. For each compound, the level of migration was calculated in % migration of solvent control.

## Results

### Safety evaluation

#### Reverse mutation assay

**Fig. 3:**
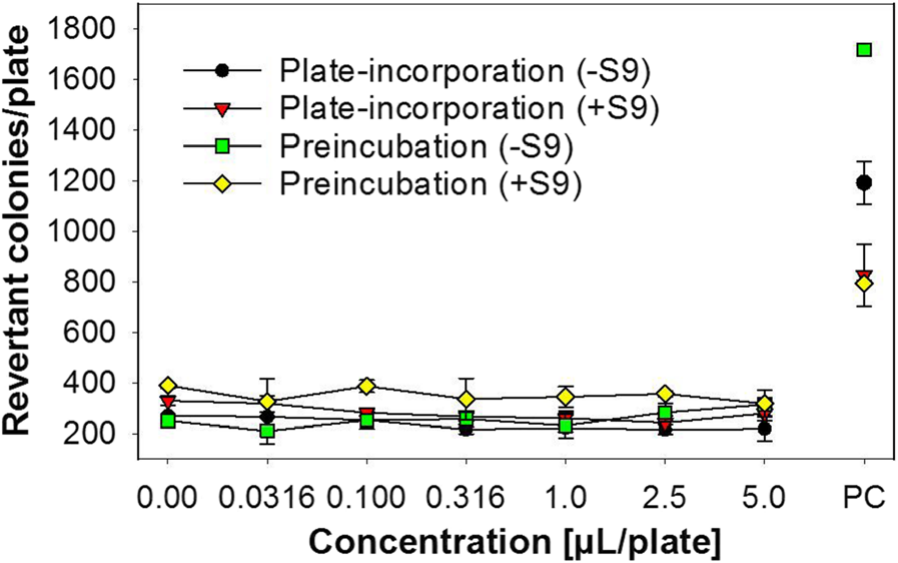
Results from Ames tests on *HNE*. Data from tester strain S. typhimurium TA102 are exemplarily presented. PC = positive controls

As part of the safety assessment of *HNE*, a bacterial reverse mutation assay was performed. In both independent experiments, no biologically relevant increase in revertant colony numbers of any of the five tester strains were observed following treatment with *HNE* at any test concentration up to the maximum exposure level recommended by OECD [22]. Metabolic activation by addition of mammalian liver microsomes did not reveal any potential to induce gene mutations. Reference mutagens induced a distinct increase of revertant colonies indicating the validity of the experiments. No biologically relevant increases in revertant colony numbers of any of the five tester strains were observed following treatment with *HNE* at any concentration level required by OECD [22]. The reference mutagens induced distinct increases of revertants (Fig. 3, see Additional file 1, 1st tab). Therefore, *HNE* is considered as non-mutagenic in this bacterial reverse mutation assay.

#### Hemolytic potential

The hemolytic activity of the *HNE* was investigated in vitro in human erythrocytes. With a hemolysis value of 62.70%, the 100 μl/ml dilution of the extract was classified as hemolytic. This value was above the threshold for hemolysis defined as > 5% in comparison with de-ionized water as positive control. In contrast, hemolysis was not observed for *HNE* at all lower concentration levels analyzed from 10 μl/ml downwards, leading to values of 2.48, 1.90, 1.68, 1.79, 2.90, and 2.21%, respectively. Standard deviations in this assay were between 0 and 0.0145 (see Additional file 1, 2nd tab). It can therefore be concluded that in the hemolysis test performed, *HNE* at concentrations up to 10 μl/ml (1:100 (v/v) dilution of *HNE*) did not induce any hemolytic effects.

### Anti-angiogenesis and tube formation

#### Matrigel angiogenesis assay

The concentration of *HNE* was predetermined with the BrdU design: *HNE* showed an anti-proliferative potency on HUVEC. The mean value of IC_50_ concentration of batches A and B of *HNE* was 120 ± 50.5 μg/ml. The reference item (Paclitaxel) is highly potent in the inhibition of cell proliferation (IC_50_ = 0.57 μg/ml). This chemotherapeutic agent belongs to the taxane group and inhibits the degradation of the microtubules and disrupts cell division.

Prior to the BrdU assay, the cytotoxic potential of *HNE* on HUVEC was evaluated by WST-1. The mean value of IC_50_ concentration of the two *HNE* batches was 288 ± 3.5 μg/ml. The reference item (Paclitaxel) was less potent in the inhibition of cellular metabolism (IC_50_ = 1238 μg/ml).

The two *HNE* batches reduced the HUVEC tube formation in the Matrigel Angiogenesis assay in a concentration-dependent manner by up to 50.1 ± 9.2% and 49.4 ± 5.3% at concentrations of 354 μg/ml and 829 μg/ml, respectively.

#### Spheroid-based cellular 3D angiogenesis assay

In the second tube formation assay, *HNE* was tested for its ability to inhibit VEGF-A induced cellular EC sprouting. The mean value of IC_50_ concentration of the two *HNE* batches was 172 ± 11.5 μg/ml. In comparison, the reference substance Sunitinib with an IC_50_ value of 11.95 μg/ml exhibited a stronger inhibitory effect.

### In vitro assessment of anti-cancer potential

#### Proliferation assays: Alamar blue assay and soft agar assay

**Fig. 4:**
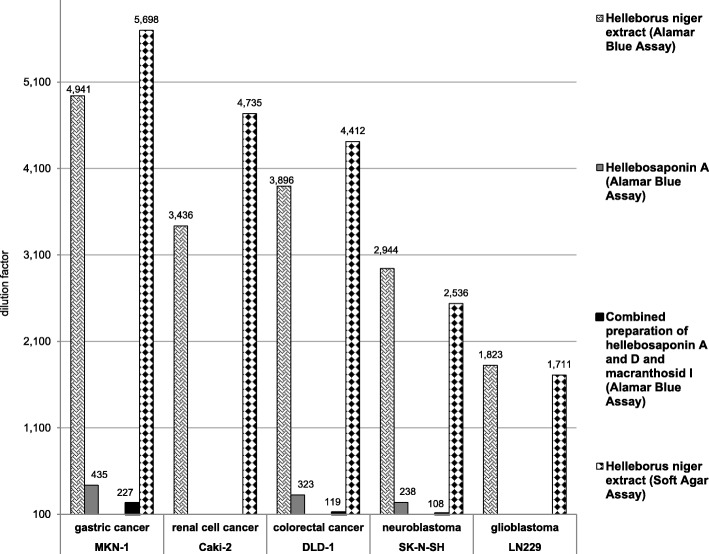
IC_50_-DF of *HNE* and its main steroidal saponins. IC_50_-dilution factor (DF) (> 1:100) of *HNE* and its main steroidal saponins hellebosaponin A and D were measured in five tumor cell lines (Caki-2: renal cell carcinoma, DLD-1: colorectal carcinoma, MKN-1: gastric carcinoma, LN 229: glioblastoma, SK-N-SH: neuroblastoma) by means of Alamar Blue and Soft Agar assays. IC_50_-DF of hellebosaponin D and macranthosid I was under 1:100. These data are not shown in this figure. The IC_50_-DF of *HNE* comprised two batches on average

The mean IC_50_-values amounted to 202–549 μg/ml in the Alamar Blue and to 176–584 μg/ml in the Soft Agar assays for the six tumor cell lines chosen (Table 2, Fig. 4). The gastric cancer cell line MKN-1 responded most sensitively and the IC_50_ of brain tumor cell lines was approximately twofold higher than on other cells. The reference compound Actinomycin-D showed an IC_50_ of 5.40 μg/ml on Caki-2 and between 1.63 μg/ml and 1.02 μg/ml on the other cell lines in the 2D design (Table 2). In the 3D model, IC_50_ values on Caki-2 were 8.79 for Actinomycin-D and between 0.31 and 1.00 μg/ml on the other cell lines (Table 2).

**Table 2:** IC_50_ concentrations (μg/ml) of *HNE* in the 2D Alamar Blue assay and 3D Soft Agar assay

	2D Alamar Blue assay					3D Soft Agar assay				
Cell type	Batch A *HNE*	Batch B *HNE*	Mean *HNE*	SD	Actinomycin-D	Batch A *HNE*	Batch B *HNE*	Mean *HNE*	SD	Actinomycin-D
Caki-2	220	430	291	105	5.40	140	430	211	145	8.79
DLD-1	210	330	257	61.5	1.58	170	340	227	35	0.99
MKN-1	170	250	202	90	1.02	130	270	176	70	0.82
LN229	460	680	549	110	1.51	440	870	584	215	0.31
SK-N-SH	260	490	340	115	1.63	330	490	394	80	1.00

Two *HNE* batches (A, B) were analyzed in comparison to Actinomycin-D in five tumor cell lines (Caki-2: renal cell carcinoma, DLD-1: colorectal carcinoma, MKN-1: gastric carcinoma, LN 229: glioblastoma, SK-N-SH: neuroblastoma) for their anti-proliferative potential in the 2D designed Alamar Blue assay and in the 3D designed Soft Agar assay. (Standard deviations of the results (growth levels relative to high control (isotonic solvent (100%)) are presented in Additional file 1)

In addition to the investigations on whole *HNE*, isolated components from *HNE* were also tested in the Alamar Blue assay. Hellebosaponin D (0.64 mg/ml) and macranthosid I (0.12 mg/ml) did not have any effect on the proliferation of the tested tumor cell lines. Hellebosaponin A (1.31 mg/ml) showed less activity than *HNE* on DLD1, SK-N-SH and MKN-1 cells (IC_50_ DF = Ø 1:332); the combined preparation of the three separate components had an even lower effect (IC_50_ DF = Ø 1:151). Hellebosaponin A and the combined preparation of the isolated saponins showed no activity on Caki-2 and LN229 cells (Table 3, Fig. 4).

**Table 3:** IC_50_ DF of the characteristic compounds at concentrations levels present in *HNE*

Cell type	Macranthosid I (0.12 mg/ml)	Hellebosaponin A (1.31 mg/ml)	Hellebosaponin D (0.64 mg/ml)	Combined preparation
Caki-2	< 1:100	< 1:100	< 1:100	< 1:100
DLD-1	< 1:100	1:323	< 1:100	1:119
MKN-1	< 1:100	1:435	< 1:100	1:227
LN229	< 1:100	< 1:100	< 1:100	< 1:100
SK-N-SH	< 1:100	1:238	< 1:100	1:108

Macranthosid I, hellebosaponin A and D are the characteristic compounds in HNE. These three compounds were isolated and investigated at concentration levels presented in *HNE* for their anti-proliferative potential by means of Alamar Blue assay. IC_50_ dilution factor (DF) of the combined preparation of all three compounds was examined in five tumor cell lines (Caki-2: renal cell carcinoma, DLD-1: colorectal carcinoma, MKN-1: gastric carcinoma, LN 229: glioblastoma, SK-N-SH: neuroblastoma) as well

#### Oris™ cell migration and invasion assay

**Fig. 5:**
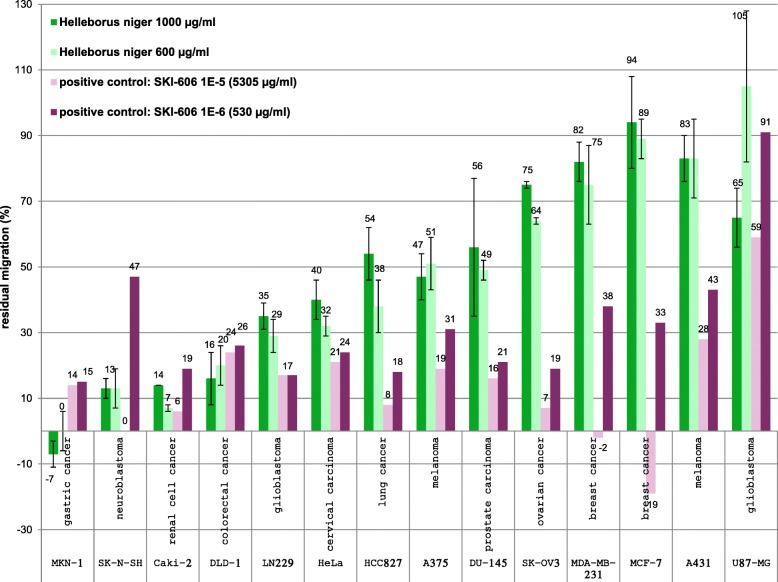
Residual migration (%) of cancer cells after treatment with *HNE.* Residual migration (%) of 14 cancer cell lines after treatment with *HNE* in two different concentrations (600 and 1000 μg/ml) compared to positive control SKI-606 in two concentrations (530 μg/ml, 5305 μg/ml) after 24–48 h depending on tumor cell lines. The cell line panel included gastric cancer (MKN-1), neuroblastoma (SK-N-SH), renal cell cancer, colorectal cancer (DLD-1), glioblastoma (LN 229 and U87-MG), cervical carcinoma (HeLa), lung cancer (HCC827), melanoma (A375 and A431), prostate carcinoma (DU-145), ovarian cancer (SK-OV3) and breast cancer (MDA-MB-231 and MCF-7). Value ranges over 100% do not mean a support of tumor cell migration but are rather due to artifact interactions in the assay

The ability to inhibit the tumor cell migration was investigated on a panel of 14 cell lines. In the concentration range of 600–1000 μg/ml, migration was inhibited by more than 80% on the neuroblastoma cell line SK-N-SH, the renal cell cancer cell line Caki-2 and the colorectal cancer cell line DLD-1. Moreover, the migration was inhibited by 60–80% on the glioblastoma cell line LN229, the cervical cancer cell line HeLa and the lung cancer cell line HCC827. Inhibition <60% was found in melanoma-, prostate carcinoma-, ovarian cancer- and breast cancer cell lines. *HNE* had a very strong (> 100%) inhibition on MKN-1 cells. The reference substance SKI-606 had a lower effect on MKN-1, SK-N-SH and Caki-2 than *HNE* and a comparable impact on DLD-1, LN229 and HeLa (Fig. 5). Standard deviations in this assay were between 0 and 23 (Fig. 5, see Additional file 1, 9th tab).

## Discussion

Although *H. niger* L. has been used as a herbal remedy for a long time, literature data still leave some uncertainties for a thorough risk assessment of the plant and extracts from it. The lactone proteoanemonin and the steroidal saponins are reported as main toxic compounds from black hellebore [1, 2]. Hence, the first part of the current research aimed to fill this gap by providing data on the preclinical safety of *HNE* used in the manufacture of medicinal products.

Genotoxicity testing is a main element in profiling the safety of herbal preparations for medical use. The Ames test is the method of choice because of its suitability to assess initial mutagenicity. The test has been shown to detect relevant genetic changes [22, 27] with a specificity of 74% compared to less than 45% in mammalian cell tests [28]. In the present study, an Ames test performed with *HNE* did not reveal any mutagenic effects neither with nor without metabolic activation by mammalian enzymes. Up to the maximum exposure level recommended by OECD [22], *HNE* did not cause gene mutations by base pair changes or frameshifts in the genome of the tester strains used in two independent experiments. Therefore, *HNE* is considered as non-mutagenic in this bacterial reverse mutation assay. Consequently, in accordance with the Committee on Herbal Medicinal Products of the European Medicines Agency (EMA’s HMPC) [27], the safety of the herbal preparation for use in traditional herbal medicine is sufficiently proven. Furthermore, results are in line with a recent publication by Schrenk et al. [29]. Therein, authors stated that neither structure-activity-relationship assessments, computer-assisted toxicity evaluation, nor comprehensive literature research revealed any evidence for a genotoxic, carcinogenic, or teratogenic potential of protoanemonin.

In the current study, the hemolytic potential of *HNE* containing steroidal saponins was investigated in vitro in human erythrocytes at different concentration levels. Hemolysis values above the threshold defined as > 5% positive control were revealed for the 100 μl/ml dilution of HNE in isotonic solution and classified as hemolytic. In contrast, hemolysis was not observed for *HNE* at concentration levels up to 10 μl/ml. It can be concluded that in the hemolysis test performed, *HNE* at concentrations up to 10 μl/ml did not induce any hemolytic effects. The level at which no hemolytic effect was observed is above the maximum bioavailable concentration expected from application of *HNE* products in humans by several orders of magnitude. Therefore, the use of *HNE* in herbal medicinal products can be considered as safe.

The aim of the second part of this investigation was to obtain a better understanding of the therapeutic potential of *HNE* in vitro used as a medicinal product in oncology. First the impact on the angiogenesis process was evaluated. Angiogenesis is mandatory for tumor progression [30, 31]. The Matrigel Angiogenesis assay monitors the ability of HUVEC to form capillary-like structures (tubes) as a prerequisite for invasive cell growth. *HNE* batches reduced the HUVEC tube formation in a concentration-dependent manner by up to 50.1 and 49.4% at concentrations of 354 and 829 μg/ml, respectively. The width of the concentration range is very large. We assume that there is an effect of saturation for *HNE* on the concentration level because the tube formation could not be stopped. In addition, on the time level the tube formation continues as the *HNE* uptake rate of the tissue is higher than the application of *HNE*. The second tube formation assay showed that 172 μg/ml *HNE* inhibited 50% of VEGF-A induced EC sprouting. This result correlates with the strong proliferation inhibition found for HUVEC in BrdU. As the VEGF-A is operating in 3D, it is more sensitive than the Matrigel Angiogenesis. The reference substance Sunitinib had a stronger inhibitory effect than *HNE* because Sunitinib inhibits all receptors for platelet-derived growth factor and vascular endothelial growth factor receptors, which play a role in tumor angiogenesis and tumor cell proliferation.

Subsequently, the anti-proliferative impact was evaluated in different tumor cell lines using cellular proliferation assays accepted in oncology for analyzing the impact of anti-cancer drugs. The most common cellular phenotypic assay is the 2D proliferation assay, which is generally regarded as a pretest to more sophisticated assays such as apoptosis or the 3D Soft Agar assay. Anchorage-independent cell growth measured in the 3D Soft Agar assay is one of the hallmark characteristics of cellular transformation and uncontrolled cell growth, with normal cells typically not capable of proliferating in semisolid matrices. 3D cell growth is more similar to the in vivo cellular environment. In the current investigations, *HNE* showed a dose-dependent anti-proliferative activity both in two- and three-dimensional extension on all five cell lines. The results of the 2D model were related to the assay in 3D design and were comparable to those from the WST-1 (Ø IC_50_ = 288 μg/ml).

The gastric cancer cell line MKN-1 and the colorectal cell line DLD-1 as well the renal cell cancer cell line Caki-2 responded most sensitively. The IC_50_ of brain tumor cell lines was approximately twofold higher than these cells, i.e. they are less sensitive towards *HNE* treatment. The reference compound Actinomycin-D, a cytotoxic antibiotic, showed a high anti-proliferative effect. It binds to Deoxyribonucleic acid (DNA) and can inhibit Ribonucleic acid- (RNA) synthesis. These results prefigure on a therapeutic potential of *HNE* not only on brain tumor or renal cell tumors, but also on gastric and colorectal cancer. This effect was confirmed in the third set of assays performed with tumor cell lines: The migration of tumor cells is considered to be an important factor for tumor spreading. Cancer cells are able to migrate, intrude into other tissues and form metastases. Therefore the Oris™ Cell Migration and Invasion assay with 14 different human tumor cell lines was performed. This assay represents an alternative to the Scratch assay because the cell seeding stoppers create a consistent detection zone to monitor 2D closure. In the concentration range of 600–1000 μg/ml, migration was inhibited by more than 80% on the colorectal cancer cell line DLD-1. The very strong inhibition (> 100%) on MKN-1 cells can be attributed to a cytotoxic effect (Alamar Blue assay: IC_50_ = 130 μg/ml). The inhibition of cell migration after treatment with *HNE* was very strong (> 90%) on SK-N-SH and strong (~ 70%) on LN229, but there was only a little effect on U87-MG glioblastoma cells, grade IV, which are refractory to chemotherapy in most cases. Value ranges over 100% do not mean a support of tumor cell migration but are rather due to artifact interactions in the assay. A very strong inhibition (90%) was also determined on the renal cancer cell line Caki-2, which is a valid proof for the current main therapeutic use [29]. Moreover, the migration was inhibited by 60–80% on the cervical cancer cell line HeLa and the lung cancer cell line HCC827, respectively. Inhibition < 60% was found in melanoma-, prostate carcinoma-, ovarian cancer- and breast cancer cell lines as well as for the reference substance SKI-606 at a concentration of 530 μg/ml on U87-MG, A431 and SK-N-SH. SKI-606 is an ATP-competitive Bcr-Abl tyrosine-kinase inhibitor with an additional inhibitory effect on SRc family kinases currently undergoing research for use in cancer treatment.

In addition to the investigations of whole *HNE*, isolated components from *HNE* were also tested in the Alamar Blue assay and showed less or no activity. The concentrations employed were representative for their content in the *HNE* batches applied. Hellebosaponin D and macranthosid I did not exert any effect on the cell proliferation. Hellebosaponin A showed about 10-fold lower activity than *HNE* on DLD1, SK-N-SH and MKN-1 cells. The combined preparation of the three separate components exhibited an even lower effect. This prefigures that other ingredients of the whole aqueous fermented extract and not primarily steroidal saponins are responsible for the anti-proliferative effects detected.

## Conclusion

*HNE* has neither genotoxic nor hemolytic potential. The current investigations verify the anti-proliferative, anti-angiogenetic and migration-inhibiting effect of *HNE*, broader than only on brain and renal cell tumors thus far associated with *HNE* treatment. The gastric cancer cell line MKN-1 responded with the greatest sensitivity, but even in DLD-1 cells (colorectal cancer) a significant anti-tumor effect was detectable, suggesting an extended field of therapeutic potential. The lower impact of the isolated characteristic steroidal saponins from *HNE* tested separately indicates that the therapeutic effect cannot be reduced to the steroidal saponins, but rather the complete aqueous fermented extract is the active principle. Concomitantly, a good safety profile was corroborated.

## Additional file

Additional file 1: Shows the results with their standard deviations (SD) in detail for all methods separated by individual tabs. **1st tab**: Genotox (Ames-test). **2nd tab**: Hemolysis-test. **3rd tab**: WST-1. **4th tab**: BrdU. **5th tab**: 2D angiogenesis assay (Matrigel Angiogenesis). **6th tab**: 3D angiogenesis assay (Spheroid-based Cellular 3D). **7th tab**: 2D proliferation assay (Alamar Blue). **8th tab**: 3D proliferation assay (Soft Agar). **9th tab**: Migration assay (ORIS™ plate). (XLSX 67 kb)

## Abbreviations

Bcr-Abl: Breakpoint cluster region Abelson murine leukemia viral oncogene homologue 1
BrdU: Bromodeoxyuridine-assay
DF: Dilution factor
DMEM: Dulbecco’s Modified Eagle’s Medium
EC: Endothelial cell
ECBM: Endothelial Cell Basal Medium
ECGM: Endothelial Cell Growth Medium
ELISA: Enzyme-linked Immunosorbent Assay
FCS: Fetal Calf Serum
GHP: German Homeopathic Pharmacopoeia
*H. niger*: *Helleborus niger* L., black hellebore, christmas rose
HMPC: Committee on Herbal Medicinal Products
*HNE*: *Helleborus niger* aqueous fermented extract
HPLC-DAD: High pressure liquid chromatography with diode array detection
HUVEC: Human Umbilical Vein Endothelial Cells
ISO: International Organization for Standardization
OD: Optical density
OECD: Organisation for Economic Cooperation and Development
ROI: Regions of interest
RPMI-1640: Rosewell Park Memorial Institute
S9: Supernatant 9000 x g
SD: Standard deviation
SRc: Cellular sarcoma
VEGF-A: Vascular Endothelial Growth Factor A
WST-1: Water Soluble Tetrazolium-assay

## Authors’ information

“Not applicable” for that section.
